# Tripartite Inca Bone in Skeletal Remains

**DOI:** 10.7759/cureus.98151

**Published:** 2025-11-30

**Authors:** Shojiro Takasu

**Affiliations:** 1 Forensic Medicine, The Jikei University, Tokyo, JPN

**Keywords:** anatomical variant, cranial variation, forensic anthropology, inca bone, interparietal bone, skeletal identification

## Abstract

The Inca bone, also known as the interparietal bone, is an anatomical variant resulting from incomplete fusion of the occipital bone during embryological development. Although its presence has been reported with varying frequencies among specific ethnic groups, its relevance in forensic anthropology remains underexplored. This report describes a human skull discovered along a riverbank, exhibiting advanced postmortem changes, including complete loss of soft tissue and dentition, as well as cranial bone destruction. Morphological analysis suggested the individual was a male over 40 years of age. Notably, a tripartite Inca bone was identified beneath the lambdoid suture. Due to its low prevalence and morphological variability, the Inca bone may serve as a useful marker in forensic identification and ancestry estimation. Accurate recognition of this variant is also important in cases of cranial trauma, where it must be differentiated from fractures. While the absence of antemortem data precluded positive identification in this case, radiological comparison with antemortem cranial imaging may facilitate identification in similar future cases. The identification of an Inca bone in forensic skeletal remains can provide meaningful anthropological and forensic insights. Forensic practitioners should remain aware of this anatomical variant during skeletal examinations to improve the accuracy of both trauma analysis and personal identification.

## Introduction

The Inca bone is an anatomical variant located on the posterior aspect of the human skull, also referred to as the interparietal bone [1,2]. It represents a persistent portion of the occipital bone that fails to fuse during embryological development.

There are notable ethnic differences in the prevalence of Inca bones, with relatively high frequencies observed among populations along the northwest coast of the Americas and in West Africa [2]. Moreover, the morphology of Inca bones exhibits considerable variation, with one or more isolated bones typically found in the upper squamous part of the occipital bone [2]. Therefore, in the field of forensic science, the presence of an Inca bone is considered to be useful for ancestry estimation and may aid in personal identification.

To the best of our knowledge, only a few studies have addressed the Inca bone in forensic contexts [3,4]. This case report describes skeletal remains featuring a prominent Inca bone and discusses its potential forensic and anthropological implications.

## Case presentation

A human skull was discovered along a riverbank by a fisherman, who promptly notified the authorities. Upon arrival, the police found no items at the scene that could assist in identifying the individual. The skull was transferred to our facility two days after its discovery.

**Figure 1:**
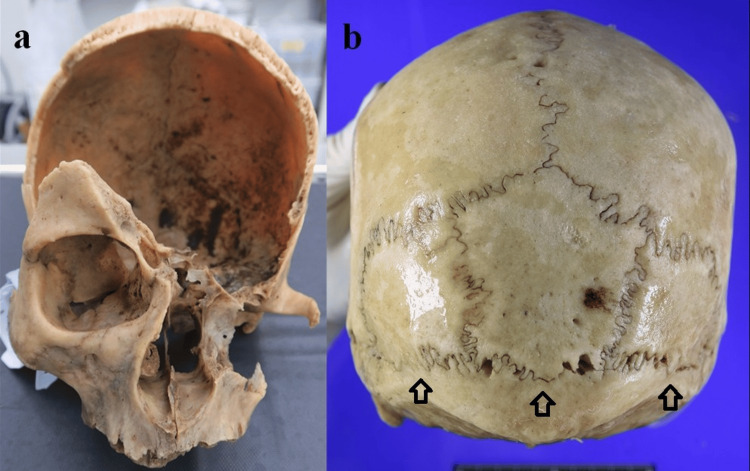
External appearance of the skull. Loss of cranial structures from the left zygomatic bone to the parietal region, accompanied by a complete absence of dental remains (a). Tripartite interparietal bone beneath the lambdoid suture (b).

The skull was completely devoid of soft tissue. Examination of the cranial remains revealed a loss of bony structures extending from the left zygomatic bone to the parietal region. Postmortem exfoliation of the teeth was noted, resulting in a complete absence of dental remains. Additionally, a tripartite interparietal bone was identified beneath the lambdoid suture (Figures 1a, 1b). The absence of the incisive and transverse palatine sutures suggested that the individual was over 40 years of age. Morphological features of the mastoid process and the external occipital protuberance were indicative of a male individual.

## Discussion

In this case, based on the morphological characteristics of the skull, the individual was estimated to be a male over 40 years of age; however, the cause of death could not be determined. Furthermore, due to the absence of soft tissue, the loss of teeth, and the destruction of cranial structures, personal identification has not yet been established.

Hanihara and Ishida classified Inca bones into six morphological types and reported their frequencies [2]. The frequency of the Inca bone type observed in the present case varies by ethnicity, ranging from 0 to 7.27% [2]. Because of their relatively low frequency and significant morphological variation, Inca bones are considered useful markers for estimating ancestry and aiding in personal identification in forensic contexts [3,5]. Additionally, in cases involving cranial trauma, it is essential to differentiate Inca bones from skull fractures [6].

In forensic science, various methods are employed for personal identification, including facial recognition, fingerprint analysis, DNA profiling, anthropological examination, and the use of medical and dental records, as well as radiological imaging [7-9]. The effectiveness of radiological comparison between antemortem and postmortem cranial CT scans for identification has been reported [9]. Moreover, Inca bones can be identified through both radiography and CT imaging [3,10]. Therefore, if antemortem cranial CT images were available in this case, personal identification might be achievable.

## Conclusions

In conclusion, the identification of an Inca bone in forensic skeletal remains offers valuable anthropological and forensic insights. Accurate recognition of this anatomical variant is essential to avoid misinterpretation as cranial trauma and can contribute to personal identification. Forensic practitioners should remain aware of the possible presence of Inca bones during skeletal analysis to improve the accuracy and reliability of their assessments.
